# Electrophysiological Correlates of Absolute Pitch in a Passive Auditory Oddball Paradigm: a Direct Replication Attempt

**DOI:** 10.1523/ENEURO.0333-18.2018

**Published:** 2018-01-08

**Authors:** Marielle Greber, Lars Rogenmoser, Stefan Elmer, Lutz Jäncke

**Affiliations:** 1 Division Neuropsychology, Department of Psychology, University of Zurich, CH-8050 Zurich, Switzerland; 2 Laboratory of Integrative Neuroscience and Cognition, Department of Neuroscience, Georgetown University Medical Center, Washington, DC 20007; 3 University Research Priority Program (URPP), Dynamics of Healthy Aging, University of Zurich, CH-8050 Zurich, Switzerland; 4 Department of Special Education, King Abdulaziz University, Jeddah 21589, Kingdom of Saudi Arabia

**Keywords:** absolute pitch, auditory, ERP, MMN, P3a, replication

## Abstract

Humans with absolute pitch (AP) are able to effortlessly name the pitch class of a sound without an external reference. The association of labels with pitches cannot be entirely suppressed even if it interferes with task demands. This suggests a high level of automaticity of pitch labeling in AP. The automatic nature of AP was further investigated in a study by Rogenmoser et al. (2015). Using a passive auditory oddball paradigm in combination with electroencephalography, they observed electrophysiological differences between musicians with and without AP in response to piano tones. Specifically, the AP musicians showed a smaller P3a, an event-related potential (ERP) component presumably reflecting early attentional processes. In contrast, they did not find group differences in the mismatch negativity (MMN), an ERP component associated with auditory memory processes. They concluded that early cognitive processes are facilitated in AP during passive listening and are more important for AP than the preceding sensory processes. In our direct replication study on a larger sample of musicians with (*n* = 54, 27 females, 27 males) and without (*n* = 50, 24 females, 26 males) AP, we successfully replicated the non-significant effects of AP on the MMN. However, we could not replicate the significant effects for the P3a. Additional Bayes factor analyses revealed moderate to strong evidence (Bayes factor > 3) for the null hypothesis for both MMN and P3a. Therefore, the results of this replication study do not support the postulated importance of cognitive facilitation in AP during passive tone listening.

## Significance Statement

A better understanding of the neural basis of absolute pitch (AP), the ability to identify a pitch without an external reference, provides valuable insights to the mechanisms of pitch processing in the human brain. Since only a tiny fraction of the population possesses AP, most previous neuroscientific research had small sample sizes. In our direct replication, we used a large sample of musicians (*n* = 104) with and without AP to confirm an intriguing finding showing that AP musicians process tones more efficiently even when not actively attending them. Using both frequentist and Bayesian analyses, we failed to replicate this effect with an identical experimental setting. This finding highlights the significance of replications and the need for large sample sizes.

## Introduction

Replications are an integral part of science. They can help estimate the size of an effect, identify the specific conditions under which it occurs, and, when successful, increase confidence in a scientific claim (Nosek et al., 2012; Brandt et al., 2014). In recent years, the low replicability of published research has become an increasing concern within neuroscience and science in general (Baker, 2016). Possible explanations for the observed low replicability include publication bias, flexibility in data analysis, and low statistical power (Munafò et al., 2017). Due to the resource-intensive data acquisition, many neuroscientific studies use small sample sizes, resulting in low power (Szucs and Ioannidis, 2017). Low power can compromise the conclusions of a study by reducing the probability of detecting a true effect, by increasing the probability that a significant finding does not reflect a true effect, and by overestimating the size of an effect (Button et al., 2013).

Acquiring data from a large sample is even more challenging for studies investigating special populations like individuals with absolute pitch (AP), the rare ability to label the pitch class (chroma) of a sound without an external reference (Takeuchi and Hulse, 1993; Zatorre, 2003; Levitin and Rogers, 2005). AP is often contrasted with relative pitch (RP), the more common ability to identify the musical interval (pitch distance) between two tones (McDermott and Oxenham, 2008). Despite its rarity, AP has received considerable scientific attention, partly because it might help understand different modes of perceptual processing and general aspects of pitch memory (Levitin and Rogers, 2005).

The neural and cognitive mechanisms underlying AP are not yet fully understood, but several studies have demonstrated that the labeling process in AP is at least in part automatic and not suppressible, even if it is disadvantageous for the task at hand (Miyazaki and Rakowski, 2002; Itoh et al., 2005; Schulze et al., 2013). The extent of this automaticity was further investigated by studies recording the electroencephalogram (EEG) during passive listening (Tervaniemi et al., 1993; Elmer et al., 2013; Matsuda et al., 2013; Rogenmoser et al., 2015). Using this approach, one can study the neurophysiological correlates of the automatic labeling process with high temporal resolution while minimizing the influence of top-down processes.

An often-used paradigm is the passive auditory oddball, in which one tone (standard) is presented more frequently than the other tones. The infrequent tones (deviants) are known to reliably elicit two frontal event-related potential (ERP) components: the mismatch negativity (MMN) and the P3a. Both ERP components are usually assessed by subtracting the standard ERP from the deviant ERP. The MMN is a negative deflection on this difference wave that peaks around 100–250 ms after stimulus onset and possibly reflects an automatic memory-based detection of change or rule violation (Picton et al., 2000; Garrido et al., 2009; Näätänen et al., 2011). While the MMN is thought to represent pre-attentive processing, the subsequently occurring positive deflection P3a has been linked to involuntary attention shifts toward unattended stimuli (Escera et al., 1998; Friedman et al., 2001; Kujala et al., 2007; Polich, 2007).

Rogenmoser et al. (2015) were the first to analyze both MMN and P3a in AP, which allowed them to study the influence of the sensory and the early cognitive processes reflected by these ERP components. They recorded EEG from 16 AP musicians and 10 non-AP musicians during a passive auditory oddball paradigm. The analysis of the MMN did not reveal any significant group differences, but AP musicians showed smaller P3a amplitudes than non-AP musicians when the deviations were larger than one semitone. The authors concluded that early cognitive processes are more efficient in AP during passive listening, whereas pre-attentive auditory processing contributes less to AP. This is in accordance with theoretical perspectives describing AP as a mainly cognitive ability (Zatorre, 2003; Levitin and Rogers, 2005).

Within small research fields like AP research, every single study has a high impact on the development of theoretical models. At the same time, the sample sizes are often small, which increases the need for replications. Rogenmoser et al. (2015) showed that AP musicians process tones differently even when not actively attending them. The extent of automaticity implied by this is both interesting and surprising. The aim of the present study was to confirm this finding in an independent and larger sample (*n* = 104). We attempted a direct replication, using the same stimuli, measures, and statistical analyses as in the original study. In addition, we calculated Bayes factors to quantify the success of the replication.

## Materials and Methods

### Participants

The current study was conducted as part of a broader research project on AP, involving multiple experiments using different imaging modalities [magnetic resonance imaging (MRI) and EEG]. Fifty-four self-reported AP possessors and 50 self-reported non-AP possessors between the age of 18 and 44 years were recruited for the current study.

All participants were professional musicians, music students, or highly-trained amateur musicians and received payment for their participation. The research protocol was approved by the local ethics committee in accordance with the Declaration of Helsinki, and all participants provided written informed consent.

None of the participants reported any past or present severe neurologic, psychiatric, or audiological disorders. Normal hearing was confirmed by pure-tone audiometry in all participants (MAICO ST 20, MAICO Diagnostic, GmbH). The two groups were matched for sex, age, handedness, age of onset of musical training, and cumulative training hours over the lifespan. Handedness was assessed by self-report and validated by the Annett Handedness Questionnaire (Annett, 1970). To control for possible between-group differences in intelligence, the Mehrfachwahl-Wortschatz-Intelligenztest (MWT-B; Lehrl, 2005) was administered. The MWT-B quantifies verbal intelligence and was shown to be a good predictor of global IQ (Lehrl et al., 1995). The musical aptitudes of the participants were assessed based on the total scores in the Advanced Measures of Music Audiation (AMMA; Gordon, 1989). To estimate musical experience in terms of age of onset of musical training and number of training hours, participants filled out an online questionnaire before taking part in the experiment. Demographical information and information on musical experience are given in Table 1.

**Table 1.** Demographics and musical experience

	AP musicians (*n* = 54)	Non-AP musicians (*n* = 50)
Sex Female Male	27 27	24 26
Age (years)	26.67 (5.49)	25.30 (4.51)
Handedness Right-handed Left-handed Both-handed	47 4 3	45 4 1
Intelligence (MWT-B)^a^	27.69 (5.10)	29.06 (4.68)
Age of onset of musical training (years)	5.93 (2.39)	6.48 (2.46)
Lifetime cumulative training (h)^b^	1.66 (1.22)	1.36 (0.96)
Musical aptitude (AMMA)^a^	66.11 (6.31)	63.22 (6.86)
Pitch-labeling test (%)	76.41 (19.55)	24.31 (19.01)

Continuous measures are given as mean (SDs in parentheses). MWT-B, Mehrfachwahl-Wortschatz-Intelligenztest; AMMA, Advanced Measures of Music Audiation.

a Raw scores.

b Units are given in 1 × 10^4^.

### Pitch-labeling test

Pitch-labeling ability was estimated using a web-based behavioral test (adapted from Oechslin et al., 2010), in which participants had to identify the pitch class and pitch height of 108 pure tones. The tones ranged from C3 to B5 (tuning: A4 = 440 Hz), lasted 500 ms, and were each presented three times in a pseudorandomized order with no tones repeated immediately in successive trials. In each trial, 2000 ms of Brownian noise was presented immediately before and after the pure tone. Answers were given by clicking on one label out of a list of all 36 possible labels (C3 to B5). Trials lasted 15,000 ms but could be terminated early by clicking on a “next” button. Pitch-labeling ability was determined by the relative frequency of correctly identified tones in terms of pitch chroma and irrespective of octave errors (Miyazaki, 1989, 1988; Takeuchi and Hulse, 1993; Deutsch, 2013).

### Stimulus material and experimental procedure

Since the current study was a direct replication, we followed the experimental procedure of the original study as closely as possible. The stimulus material and the code for stimulus presentation were identical to those used in the original study. The auditory stimuli consisted of five piano tones with different fundamental frequencies. Three of the tones were in tune (C4 = 264 Hz, A4 = 440 Hz, A♭4/G#4 = 416 Hz) and two of the tones were mistuned (1/4-semitone deviation of A♭4/G#4 = 422 Hz, 1/10-semitone deviation of A4 = 438 Hz). All piano tones were recorded as 16-bit stereo files and had a duration of 200 ms with 5-ms rise and fall time. Their overall amplitude was normalized to ensure equal intensities.

During EEG recording, the auditory stimuli were presented binaurally with HiFi headphones (Sennheiser, HD 25-1, 70 Ω, Ireland) at a sound pressure level of 70 dB. Stimulus presentation was controlled by the Presentation software (version 18.1, RRID:SCR_002521). The participants were instructed to watch a silent black and white film and to ignore the simultaneously presented auditory stimuli. This passive listening experiment consisted of five blocks, presented in a random order across participants. In each block, one of the five piano tones was presented more frequently (420 times, occurrence probability = 60%; standard tone) than the other four (70 times each, occurrence probability = 10%; deviant tones). Each piano tone served as standard tone in one block and as deviant tone in all other blocks. As the EEG analyses of the original study, we focused on the blocks with standard tones of 440 Hz (block A) and of 264 Hz (block C). In these blocks, deviation magnitude increased or decreased unambiguously. Therefore, it was possible to test the effect of deviation magnitude on the EEG signal. Table 2 provides an overview of the study design. Presentation of the stimuli was pseudorandomized in each block. To establish a stable memory trace (Näätänen and Winkler, 1999), the first 15 tones were standards. For the remaining trials, deviants were always followed by at least one standard tone, and at least two different deviants were inserted before the same deviant could appear again. The interstimulus interval between the tones was fixed to 550 ms. The entire EEG recording lasted around 45 min.

**Table 2.** Study design

	Standard tone	Deviant tones			
Block A	440 Hz	438 Hz	422 Hz	416 Hz	264 Hz
Block C	264 Hz	416 Hz	422 Hz	438 Hz	440 Hz

Deviant tones are listed from left to right according to increasing deviation magnitude.

### EEG recording and preprocessing

EEG data were recorded with a sampling rate of 1000 Hz and an online bandpass filter of 0.1–100 Hz using a BrainAmp amplifier (Brainproducts). Thirty-two silver/silver-chloride electrodes were placed according to a subset of the 10/10 system, and an electrode on the tip of the nose was used as the reference. Electrode impedance was kept below 10 kΩ by applying an electrically conductive gel.

**Figure 1.**
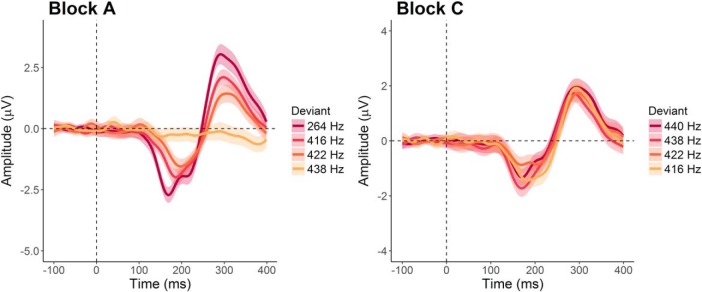
Grand averages of the difference waves (deviant ERP minus standard ERP). ERPs from the fronto-central pooling of electrodes were averaged over all participants for each deviation condition. The lines represent the means, the shaded areas indicate 95% within-subject confidence intervals. Darker colors illustrate larger deviation magnitudes. In block A (standard tone 440 Hz), amplitudes increase with larger deviation magnitude. In block C (standard tone 264 Hz), no such clear relationship can be observed.

**Figure 2.**
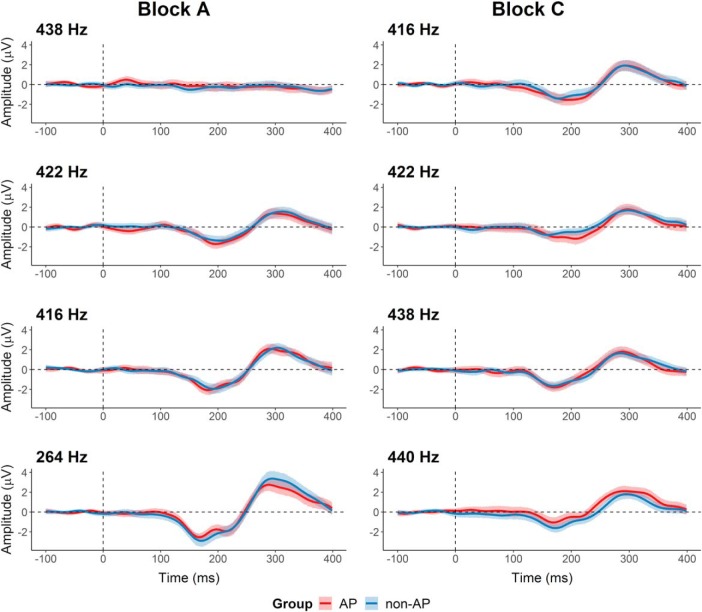
Grand averages of the difference waves (deviant minus standard) for AP (in red) and non-AP (in blue) musicians. Deviation magnitude increases from top to bottom. The lines represent the group means, the shaded areas represent the 95% between-subject confidence interval.

Preprocessing of the EEG data was conducted with the BrainVision Analyzer software package (version 2.1, https://www.brainproducts.com/, RRID:SCR_002356). Data were filtered offline with a bandpass filter of 1–20 Hz (48 dB/octave) and a notch filter of 50 Hz. Eye movement artifacts (eye blinks and saccades) were corrected using an independent component analysis (ICA; Jung et al., 2000), and noisy channels were interpolated. Remaining artifacts were removed using an automatic raw data inspection algorithm when a voltage gradient criterion of 50 µV/ms, an amplitude criterion of ±100 µV, or a low activity criterion of 0.5 µV/100 ms was exceeded. After preprocessing, the EEG signal was divided into segments of 500 ms (–100–400 ms from stimulus onset). These segments were baseline corrected (–100–0 ms) and averaged to ERPs. To compute difference waves, the ERPs evoked by the five standard tones were subtracted from the ERPs evoked by the physically identical deviants presented in the two blocks of interest (block A and block C). The grand averages of the difference waves for each deviant over all participants are shown in Figure 1. In Figure 2, the grand averages are presented separately for each group.

**Figure 3.**
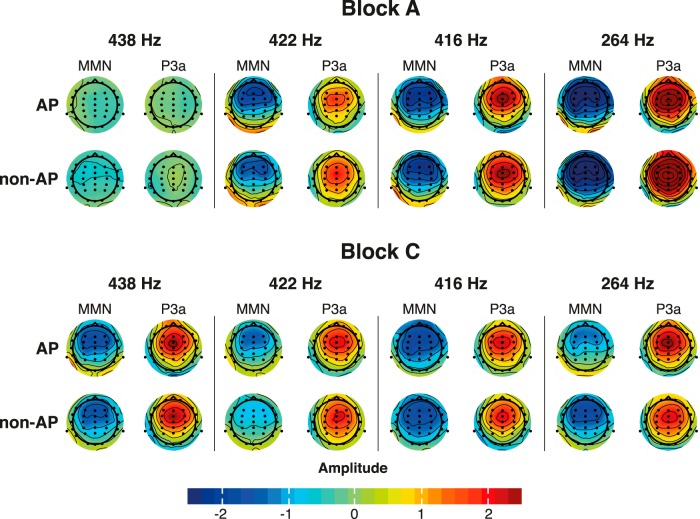
Voltage distributions over the scalp for the MMN and P3a for each group and each deviant in block A (standard tone 440 Hz) and block C (standard tone 264 Hz). Topographies are shown at the time point of the peak according to the grand average of the specific deviation condition and group. Deviation magnitude increases from left to right. Both MMN and P3a are maximally expressed at fronto-central electrode sites.

We extracted peak values of the resulting difference waves for the MMN and P3a from a pooling of nine frontal and central electrodes (F3, Fz, F4, FC3, FCz, FC4, C3, Cz, C4). In the original study, both ERP components elicited maximal amplitudes over these electrodes, and a similar voltage distribution could be observed in the data of the current replication study (Fig. 3; the topographical maps were created using code from the R package *EEGutils*; Craddock, 2018). Peaks were selected using an automatic peak detection algorithm and verified by visual inspections.

### Statistical analyses

All statistical analyses were conducted in R (version 3.4.3; https://www.r-project.org, RRID:SCR_001905). To compare the groups in terms of demographics and musical experience, we applied Welch’s *t* tests. Effect sizes for *t* tests are given in Cohen’s *d* (Cohen, 1988).

For statistical analyses of the peak amplitudes and latencies, we replicated the null hypothesis statistical testing (NHST) of the original paper (replication analyses) and additionally performed Bayes factor analyses (exploratory Bayesian analyses).

In the replication analyses, a two-way mixed ANOVA with two levels of group (AP and non-AP) and four levels of deviation (four deviants) was computed separately for each ERP component and each block of interest using the R package *ez* (version 4.4.0; https://cran.r-project.org/web/packages/ez/index.html); *p* values and degrees of freedom were adjusted using Greenhouse–Geisser correction when Mauchly’s test revealed non-sphericity. For the ANOVAs, generalized eta-squared (η^2^_G_) is reported as the effect size estimate (Bakeman, 2005). Additionally, we report Cohen’s *d* for the main effect of group (Cohen, 1988). As in the original study, results with *p* ≤ 0.05 are termed significant.

### Bayes factors

Using NHST provides direct comparability with the original study. However, because NHST only allows to reject the null hypothesis (H0), but not the alternative (H1), non-significant results cannot differentiate between insensitive data and evidence in favor of H0. To decide whether a replication was successful or not, a quantification of null results is especially useful. Contrary to NHST, Bayes factors allow such conclusions on whether the evidence supports H0, the evidence supports H1, or the evidence is ambiguous (Rouder et al., 2009; Dienes, 2011, 2014; Lee and Wagenmakers, 2013). Bayes factors express the ratio between the likelihood of the data under one hypothesis (e.g., H0) relative to another hypothesis (e.g., H1). A Bayes factor BF01 of 10 (or the inverse $\frac{1}{{BF}_{01}}$= BF_10_ = 0.1) can be directly interpreted as the data being 10 times more likely to occur under H0 compared to H1. As a consequence, Bayes factors are well suited to interpret non-significant results (Dienes, 2014) and to quantify the success of a replication (Verhagen and Wagenmakers, 2014; Anderson and Maxwell, 2016).

We calculated Bayes factors using the default Cauchy priors (scaling factor *r* = 0.707) as implemented in the *BayesFactor* package in R (version 0.9.12-4.2; https://cran.r-project.org/web/packages/BayesFactor/index.html) with 100,000 iterations. Priors were not based on the effect sizes reported in the original study because small samples often result in inflated effect size estimates (Ioannidis, 2008; Button et al., 2013; Halsey et al., 2015). However, to ensure the robustness of our results, we additionally tested a range of priors (i.e., *r* = 0.50, *r* = 1.00, *r* = 1.20), and the results supported the same main conclusions.

Paralleling the replication analyses, we performed Bayesian ANOVAs (BANOVA; Rouder et al., 2017) on the peak amplitudes and latencies separately for each ERP component in each block. Bayes factors of interaction effects were assessed by comparing the full model (group + deviation + group × deviation + subject) to the model without the interaction effect (group + deviation + subject).

To facilitate interpretation, we report *BF_10_* when Bayes factors favored the alternative hypothesis and *BF_01_*($\frac{1}{{BF}_{10}}$) when Bayes factors favored the null hypothesis. Following Jeffreys (1961; edited by Lee and Wagenmakers, 2013)’s terminology, a Bayes factor between 1 and 3 is considered anecdotal evidence, between 3 and 10 moderate evidence, between 10 and 30 strong evidence, between 30 and 100 very strong evidence, and above 100 extreme evidence for the respective hypothesis.

## Results

### Demographics and behavioral data

**Figure 4.**
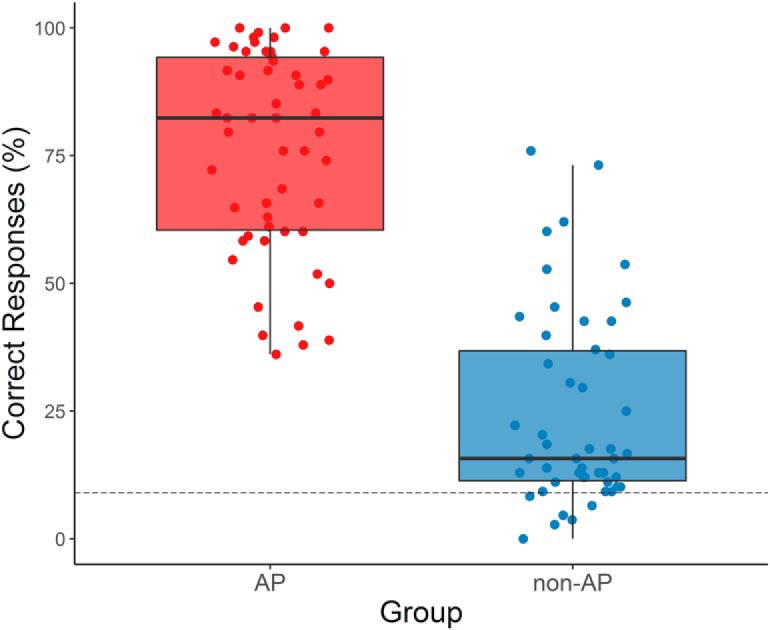
Performance in the pitch-labeling test for AP and non-AP musicians. Octave errors were treated as correct answers, resulting in a chance level of 8.33% (dashed line). AP musicians are depicted in red, non-AP musicians in blue. AP musicians performed significantly better than non-AP musicians (*t*_(101.75)_ = 13.77, *p* < 0.001, *d* = 2.70).

Welch’s *t* tests did not reveal any significant group differences in age (*t*_(100.58)_ = 1.39, *p* = 0.17, *d* = 0.27), intelligence (*t*_(101.99)_ = –1.43, *p* = 0.15, *d* = 0.28), age of onset of musical training (*t*_(100.89)_ = –1.16, *p* = 0.25, *d* = 0.23), and cumulative musical training hours over the lifespan (*t*_(99.49)_ = 1.41, *p* = 0.16, *d* = 0.27). However, the two groups differed in musical aptitude (*t*_(99.41)_ = 2.23, *p* = 0.028, *d* = 0.44), and AP musicians performed significantly better in the pitch-labeling test (*t*_(101.75)_ = 13.77, *p* < 0.001, *d* = 2.70; Fig. 4).

### Electrophysiological data: replication analyses

**Figure 5.**
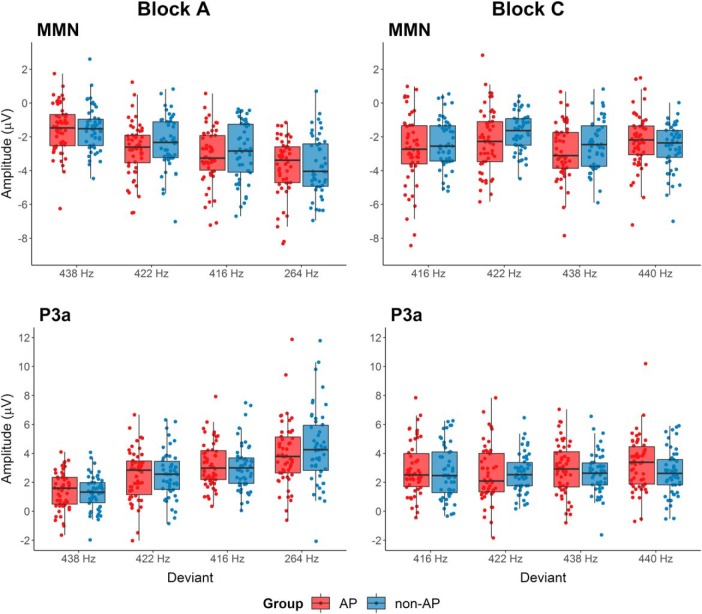
MMN and P3a amplitudes of musicians with AP and without AP (non-AP) for all deviation conditions in block A (standard tone 440 Hz) and block C (standard tone 264 Hz). Deviants are ordered from left to right according to increasing deviation magnitude. Amplitudes of AP musicians are shown in red, amplitudes of non-AP musicians are shown in blue.

The analyses of the MMN amplitudes and latencies showed similar results as in the original study. The original study reported main effects of deviation for MMN amplitudes and latencies, but only in block A. In the present study, we found a significant main effect of deviation on MMN amplitudes in both block A (*F*_(2.90,296.15)_ = 45.60, *p* < 0.001, η^2^_G_ = 0.21) and block C (*F*_(2.92,297.71)_ = 4.28, *p* = 0.006, η^2^_G_ = 0.03). However, the generalized eta-squared indicated that the effect in block C was small and comparable to the one obtained in the original study (η^2^_G_ = 0.04). Additionally, as visible in Figures 1, 5, the amplitudes did not consistently get larger with increasing deviation magnitude in block C. As in the original study, the analysis did not reveal any significant effects of group (block A: *F*_(1,102)_ = 0.45, *p* = 0.51, η^2^_G_ = 0.002, *d* = 0.08; block C: *F*_(1,102)_ = 1.52, *p* = 0.22, η^2^_G_ = 0.005, *d* = 0.14) or significant interactions for MMN amplitudes (block A: *F*_(2.90,296.15)_ = 0.52, *p* = 0.66, η^2^_G_ = 0.003; block C: *F*_(2.92,297.71)_ = 1.87, *p* = 0.14, η^2^_G_ = 0.01).

A similar pattern was found for MMN latencies. There was a significant main effect of deviation in block A (*F*_(2.52,256.66)_ = 4.99, *p* = 0.004, η^2^_G_ = 0.03) and block C (*F*_(2.86,291.60)_ = 7.60, *p* < 0.001, η^2^_G_ = 0.04), but effect sizes were small. The main effects of group (block A: *F*_(1,102)_ = 0.01, *p* = 0.94, η^2^_G_ < 0.001, *d* = 0.008; block C: *F*_(1,102)_ = 0.42, *p* = 0.52, η^2^_G_ = 0.002, *d* = 0.08) and the interactions (block A: *F*_(2.52,256.66)_ = 0.78, *p* = 0.48, η^2^_G_ = 0.005; block C: *F*_(2.86,291.60)_ = 0.80, *p* = 0.49, η^2^_G_ = 0.004) did not reach significance.

The main result reported in the original study were reduced P3a amplitudes in AP musicians compared to non-AP musicians. P3a latencies were not evaluated in the original study but are reported here for completeness. In line with the original study, the replication analyses showed a significant main effect of deviation on P3a amplitudes in block A (*F*_(2.63,268.46)_ = 55.02, *p* < 0.001, η^2^_G_ = 0.25), but not in block C (*F*_(2.87,292.91)_ = 1.39, *p* = 0.25, η^2^_G_ = 0.007). However, contrary to the original study, we did not find any significant main effects of group (block A: *F*_(1,102)_ = 0.08, *p* = 0.78, η^2^_G_ = 0.002, *d* = 0.03; block C: *F*_(1,102)_ = 1.19, *p* = 0.28, η^2^_G_ = 0.006, *d* = 0.15) or interaction effects (block A: *F*_(2.63,268.46)_ = 0.92, *p* = 0.42, η^2^_G_ = 0.005; block C: *F*_(2.87,292.91)_ = 1.14, *p* = 0.33, η^2^_G_ = 0.005) for P3a amplitudes (Fig. 5).

The analysis of P3a latencies also revealed a significant main effect of deviation in block A (*F*_(2.22,226.56)_ = 5.58, *p* = 0.003, η^2^_G_ = 0.04), but no significant main effect of group (*F*_(1,102)_ = 0.09, *p* = 0.77, η^2^_G_ < 0.001, *d* = 0.03) and no interaction (*F*_(2.22,226.56)_ = 0.50, *p* = 0.63, η^2^_G_ = 0.003). In block C, there was no significant main effect (deviation: *F*_(2.87,292.44)_ = 1.58, *p* = 0.20, η^2^_G_ = 0.009; group: *F*_(1,102)_ = 0.05, *p* = 0.82, η^2^_G_ < 0.001, *d* = 0.03) or interaction (*F*_(2.87,292.44)_ = 0.43, *p* = 0.72, η^2^_G_ = 0.002).

### Electrophysiological data: exploratory Bayesian analyses

Replication analyses of MMN and P3a amplitudes yielded non-significant results for all group comparisons. To better distinguish between insensitive evidence, evidence for the alternative hypothesis, and evidence for the null hypothesis, we computed Bayes factors.

For MMN amplitudes, the Bayes factors mostly mirrored the results from the replication analyses. In block A, we obtained extreme evidence for an effect of deviation (BF_10_ = 7.32 × 10^21^), moderate evidence for the absence of an effect of group (BF_01_ = 5.93) and strong evidence for the absence of an interaction effect (BF_01_ = 21.52). In block C, evidence for an effect of deviation was less strong than in block A (BF_10_ = 3.25). Further, Bayes factors showed moderate evidence that there was no group difference (BF_01_ = 3.70) and no interaction (BF_01_ = 3.92).

As in the replication analyses, results for the MMN latencies were similar to those obtained for MMN amplitudes. Bayes factors provided evidence for the existence of a difference between deviants in block A (BF_10_ = 9.36) and block C (BF_10_ = 242.91), but not for differences between groups (block A: BF_01_ = 7.17; block C: BF_01_ = 5.10) or for an effect of interaction (block A: BF_01_ = 15.28; block C: BF_01_ = 15.77).

The replication analyses of P3a amplitudes revealed a significant effect of deviation in block A. All other effects did not reach significance. Bayes factors strongly supported the existence of a difference between deviants in block A (BF_10_ = 2.06 × 10^26^), but not in block C (BF_01_ = 15.86). In terms of group differences, there was moderate evidence for the null hypothesis in both block A (BF_01_ = 7.32) and block C (BF_01_ = 3.14). Bayes factors also strongly favored the null hypothesis regarding the interaction (block A: BF_01_ = 13.40; block C: BF_01_ = 10.40).

For P3a latencies, there was strong evidence for an effect of deviation in block A (BF_10_ = 26.64). For all other effects, Bayes factors provided support for the null hypothesis in both block A (group: BF_01_ = 7.29; interaction: BF_01_ = 22.07) and block C (deviation: BF_01_ = 15.86; group: BF_01_ = 6.30; interaction: BF_01_ = 10.40).

### Electrophysiological data: exploratory subgroup analyses

The sample of the present study differed from the sample of the original study in three main ways: First, our sample was quite evenly balanced in terms of gender while the original study investigated predominantly female subjects. This might have influenced the results as females have previously been shown to have larger P3a amplitudes than males (visual paradigm, Conroy and Polich, 2007). Second, there was no overlap between the two groups in the pitch-labeling scores in the original study, but there is an overlap in our sample. Third, there was a small but significant difference in musical aptitude (AMMA) between groups in the present study.

Since all these sample differences could account for the differences in the results, we conducted additional subgroup analyses for the P3a amplitude. One subgroup analysis was performed on just the female participants of our study (n_AP_ = 27, n_non-AP_ = 24). A second subgroup analysis was performed on the third of the participants with the lowest pitch-labeling scores (<31.79%, *n* = 35) and the third of the participants with the highest pitch-labeling scores (>72.83%, *n* = 35). This allowed us to check whether the absence of the AP effect on the P3a was due to the more heterogenous groups in the present study. A third subgroup analysis corresponded as closely as possible to the original study in terms of pitch-labeling scores and sample size: only participants with scores <10% (*n* = 9) and >93% (*n* = 15) entered this analysis. Finally, we also performed an analysis of covariance (ANCOVA) with the AMMA score as covariate to test whether the between-group difference in musical aptitude influenced the result.

For the subgroup of females only, analysis of the P3a amplitude revealed an effect of deviation in block A (*F*_(2.75,134.94)_ = 21.83, *p* < 0.001, η^2^_G_ = 0.23, BF_10_ = 1.13 × 10^10^) but no effect of group (*F*_(1,49)_ = 0.20, *p* = 0.66, η^2^_G_ = 0.001, *d* = 0.063, BF_01_ = 4.95) or an interaction effect (*F*_(2.75,134.94)_ = 0.35, *p* = 0.77, η^2^_G_ = 0.004, BF_01_ = 12.72). No significant effect was found in block C (group: *F*_(1,49)_ = 0.29, *p* = 0.59, η^2^_G_ = 0.003, *d* = 0.11, BF_01_ = 3.43; deviation: *F*_(2.89,141.73)_ = 0.68, *p* = 0.56, η^2^_G_ = 0.007, BF_01_ = 17.61; interaction: *F*_(2.89,141.73)_ = 0.35, *p* = 0.78, η^2^_G_ = 0.003, BF_01_ = 12.74).

Similarly, the analysis with the lowest and highest performing third of participants showed an effect of deviation in block A (*F*_(2.63,178.59)_ = 38.39, *p* < 0.001, η^2^_G_ = 0.27, BF_10_ = 9.96 × 10^17^) but no effect of group (*F*_(1,68)_ = 0.04, *p* = 0.83, η^2^_G_ < 0.001, *d* = 0.09, BF_01_ = 5.18) or an interaction effect (*F*_(2.63,178.59)_ = 0.38, *p* = 0.74, η^2^_G_ = 0.003, BF_01_ = 18.79). Again no significant effects were observed in block C (group: *F*_(1,68)_ = 2.72, *p* = 0.11, η^2^_G_ = 0.02, *d* = 0.35, BF_10_ = 1.50; deviation: *F*_(2.78,188.84)_ = 0.93, *p* = 0.42, η^2^_G_ = 0.007, BF_01_ = 18.74; interaction: *F*_(2.78,188.84)_ = 2.42, *p* = 0.072, η^2^_G_ = 0.02, BF_01_ = 2.88).

Likewise, with even more extreme groups (<10% and >93% pitch-labeling performance), there was an effect of deviation in block A (*F*_(2.54,55.91)_ = 24.34, *p* < 0.001, η^2^_G_ = 0.44, BF_10_ = 5.97 × 10^9^) but no other effect in block A (group: *F*_(1,22)_ = 0.03, *p* = 0.86, η^2^_G_ < 0.001, *d* = 0.03, BF_01_ = 3.62; interaction: *F*_(2.54,55.91)_ = 0.64, *p* = 0.57, η^2^_G_ = 0.02, BF_01_ = 4.61) or block C (group: *F*_(1,22)_ = 2.68, *p* = 0.12, η^2^_G_ = 0.06, *d* = 0.55, BF_01_ = 1.03; deviation: *F*_(2.67,58.74)_ = 1.22, *p* = 0.31, η^2^_G_ = 0.02, BF_01_ = 4.61; interaction: *F*_(2.67,58.74)_ = 0.91, *p* = 0.43, η^2^_G_ = 0.02, BF_01_ = 2.94).

The ANCOVA with the AMMA score as covariate on the full sample revealed similar results: an effect of deviation in block A (*F*_(2.63,268.46)_ = 55.02, *p* < 0.001, η^2^_G_ = 0.25) and no other effects neither in block A (group: *F*_(1,102)_ = 0.04, *p* = 0.85, η^2^_G_ < 0.001; interaction: *F*_(2.63,268.46)_ = 0.92, *p* = 0.42, η^2^_G_ = 0.01) nor in block C (group: *F*_(1,102)_ = 1.95, *p* = 0.17, η^2^_G_ = 0.009; deviation: *F*_(2.87,292.91)_ = 1.39, *p* = 0.25, η^2^_G_ = 0.007; interaction: *F*_(2.87,292.91)_ = 1.14, *p* = 0.33, η^2^_G_ = 0.006).

We also performed an ANCOVA on the subgroup of participants with comparable sample size and pitch-labeling scores as in the original study. Again, we found an effect of deviation in block A (*F*_(2.54,55.91)_ = 24.34, *p* < 0.001, η^2^_G_ = 0.44) but no other effects in either block A (group: *F*_(1,22)_ = 0.04, *p* = 0.85, η^2^_G_ < 0.001; interaction: *F*_(2.54,55.91)_ = 0.64, *p* = 0.57, η^2^_G_ = 0.02) or block C (group: *F*_(1,22)_ = 3.81, *p* = 0.064, η^2^_G_ = 0.08; deviation: *F*_(2.67,58.74)_ = 1.22, *p* = 0.31, η^2^_G_ = 0.03; interaction: *F*_(2.67,58.74)_ = 0.91, *p* = 0.43, η^2^_G_ = 0.02).

## Discussion

In the present study, we attempted to replicate Rogenmoser et al. (2015)’s finding of electrophysiological group differences between AP and non-AP musicians during passive listening. Rogenmoser et al. (2015) investigated the automatic nature of AP by recording EEG during a passive auditory oddball paradigm. By analyzing MMN and P3a, they intended to assess the contribution of both pre-attentive (as reflected by the MMN) and more cognitive processes (as reflected by the P3a) in AP. To compare the tone processing between AP and non-AP musicians under different deviation conditions, they applied a paradigm with multiple tuned and mistuned deviants. In line with previous research (Tervaniemi et al., 1993; Matsuda et al., 2013, condition with tuned tones), they did not find any significant group differences in the MMN. In contrast, Rogenmoser et al. (2015) observed smaller P3a amplitudes in AP musicians. This group difference was only found in conditions in which the deviation magnitude was larger than one semitone (264-Hz deviant in block A and all deviants in block C), suggesting that AP musicians process between-pitch but not within-pitch categories differentially than non-AP musicians. Because the P3a has been associated with an early reallocation of attention (Escera et al., 1998; Friedman et al., 2001; Kujala et al., 2007; Polich, 2007), the smaller amplitudes in AP musicians were interpreted as an indication for more efficient cognitive tone processing in AP. The authors concluded that the “P3a component turned out to be a specific marker for AP” (Rogenmoser et al., 2015).

In the current direct replication study, we found no significant group differences in the MMN, confirming the results of the original study. However, and most critically, there were also no significant group differences in the P3a. Additional Bayes factor analyses revealed that the data are more likely under the null hypothesis, implying that AP and non-AP musicians’ tone processing, as indicated by MMN and P3a peak amplitudes and latencies, does not differ during passive listening. Thus, our results challenge the view of cognitive facilitation in AP during passive listening.

In passive auditory oddball paradigms, the MMN typically occurs in response to a change (deviation) in auditory stimulation within a sequence of repeated stimuli (standard tone). The main generator of the MMN is located in the auditory cortex (for review, see Näätänen et al., 2007), where the repeated presentation of a stimulus potentially causes the formation of a short-term memory trace (Näätänen and Winkler, 1999). The MMN is generated when a new auditory input differs from the representation in this sensory memory trace. Because this mismatch detection process does not require that the stimuli are attended, it is thought to be automatic (Sussman et al., 2003; Paavilainen et al., 2007). Accordingly, the MMN is considered an objective measure of auditory discrimination accuracy (Näätänen et al., 2007). Consistent with this view, it has been shown that the amplitude of the MMN increases when discrimination performance improves through training (Näätänen et al., 1993; Menning et al., 2000; Atienza et al., 2002). The MMN amplitude also correlates more generally with behavioral discrimination accuracy (Novak et al., 1990; Näätänen et al., 1993). Similarly, the MMN is also influenced by the deviation magnitude, with larger, and therefore more salient, deviations evoking larger amplitudes and shorter latencies (Sams et al., 1985; Berti et al., 2004; Novitski et al., 2004).

The original study reported an effect of deviation magnitude for block A but not for block C. The authors provided a possible explanation that in block C, all deviants were clustered around an extreme deviation level, with a distance between eight and nine semitones from the standard tone. Consequently, all deviants were probably equally easy to detect. In accordance with the original study, our results showed larger MMN amplitudes and shorter MMN latencies for larger deviations in block A. In block C, the effect also reached significance, but like in the original study, amplitudes did not unambiguously increase with deviation magnitude (compare Fig. 3), suggesting a context effect in this specific block.

More importantly, we also replicated the result of non-significant group differences between the AP and non-AP musicians in MMN measures. The Bayes factor analysis additionally provided support for the null hypothesis. Thus, our data were more likely under the hypothesis that there were no differences in the MMN amplitudes and latencies between the two groups than under the H_1_. Our results are not only consistent with the original study but also with other previous research. Using tuned and mistuned pure tones and piano tones, Tervaniemi et al. (1993) did not find group differences between AP and non-AP musicians in MMN amplitudes and latencies. In Matsuda et al. (2013)’s study, MMN amplitudes of AP and non-AP musicians did also not differ for tuned tones, but AP musicians showed larger MMN amplitudes for mistuned tones. However, this effect might have been influenced by the fact that their AP musicians were musically more experienced than the non-AP musicians. Previous research has shown that musical experience can increase MMN amplitudes (Koelsch et al., 1999; Putkinen et al., 2014), specifically in response to mistuned tones (Tervaniemi et al., 2014).

Because the MMN is associated with a passive discrimination process, Tervaniemi et al. (1993) concluded from their results that “pitch naming and discrimination are based on different brain mechanisms.” This coincides with results from behavioral studies showing that pitch-labeling accuracy is not correlated with behavioral pitch-discrimination accuracy (Sergeant, 1969; Fujisaki and Kashino, 2002). Thus, evidence from both behavioral and electrophysiological data suggests that AP does not simply rely on refined pitch discrimination.

In passive auditory oddball paradigms, the MMN is often followed by the P3a, a subcomponent of the P300. Both components have been proposed to play a role in the reallocation of attention to unattended stimuli (Näätänen, 1990; Escera et al., 2000; Kujala et al., 2007), with the processes underlying MMN probably initiating the attention switching and the P3a directly reflecting it. The P3a is affected by the magnitude of deviation in similar ways as the MMN (Berti et al., 2004). As for the MMN, the original study found such a deviation modulation only in block A, probably again due to the more extreme deviation levels in block C. The present study successfully replicated these results. In block A, P3a amplitudes increased and P3a latencies decreased with increasing deviation, and as in the original study, no similar effect was observed in block C. Future studies should more systematically investigate this dependence on specific contexts.

Although the modulation of the MMN and P3a as a function of deviation magnitude is an interesting aspect of general pitch processing, the main finding of the original study was the reduced P3a amplitudes in AP musicians. This result was compared to findings from the parietal P3b, another subcomponent of the P300, which is elicited in active oddball paradigms and often called P300 in these studies. The P3b has been linked to working memory updating (for review, see Kok, 2001; Polich, 2007) and has been investigated more thoroughly in AP research than the P3a. The first study to detect differences in ERPs during pitch processing reported the absence of a P3b in individuals with AP (Klein et al., 1984). This was regarded as an indication that individuals with AP did not need to update their auditory working memory during the task because their pitch representations are permanent. Subsequently, some studies replicated the absence or diminution of P3b amplitudes in AP (Hantz et al., 1992; Wayman et al., 1992; Crummer et al., 1994), but others did not (Hantz et al., 1995; Hirose et al., 2002). . This inconsistency was shown to be caused by differential pitch-processing strategies (RP or AP) employed by the participants based on the specific task instructions, the task difficulty, and the individual level of AP (Bischoff Renninger et al., 2003).

Individual differences in listening strategies could explain why we did not replicate the effect of AP on the P3a. However, this seems rather unlikely as the use of top-down strategies was controlled with the help of a distractor task (watching a silent film) in both the original and the replication study. Given how unreliable the effect of AP on ERPs is even in active tasks, we believe it is more plausible that the differences in passive pitch processing are too subtle to be reliably detectable with ERP peak measures. Alternatively, it could also be speculated that the pitch labeling is only initiated when actively attending the auditory stimuli or when performing a labeling-related task (e.g., bimodal Stroop task; Akiva-Kabiri and Henik, 2012). Compelling evidence for an automatic pitch-labeling process comes from behavioral studies, in which the auditory stimuli had to be attended to solve the task. For instance, individuals with AP performed poorer in auditory Stroop tasks when they heard sung tone names and were instructed to repeat the syllable while ignoring the pitch it was sung in (Miyazaki, 2004; Itoh et al., 2005; Schulze et al., 2013). AP also hindered performance in a RP task, in which participants had to compare a visual notation with the auditory presentation of a melody (Miyazaki and Rakowski, 2002). Further evidence for the automaticity of pitch labeling was provided by neuroscientific studies that observed differential electrophysiological or hemodynamic responses in AP musicians during attentive listening (Zatorre et al., 1998; Itoh et al., 2005). Contrary to these studies, in the present study, participants were instructed to focus their attention on a silent film and to ignore the auditory stimuli altogether. AP musicians can label tones fast and effortlessly, but they may not necessarily do so under all circumstances. Apart from the specific task, also other situational factors like stress and fatigue might influence pitch-labeling performance and pitch-labeling automaticity. Additionally, it is also possible that there are considerable interindividual differences in the level of automaticity of AP per se. Future studies will hopefully uncover the role of such influences on this extraordinary ability and its neural underpinnings in more detail.

Although this study could not demonstrate a cognitive facilitation in AP during passive listening, we believe our results do not challenge existing cognitive theories of AP, like the two-component model (Levitin, 1994). The two-component model focuses on the use of long-term pitch memory representations and their association with labels in AP. This mechanism in turn poses less demands on working memory in some tasks than using RP (Klein et al., 1984; Itoh et al., 2005; Schulze et al., 2009). In contrast to these mnemonic processes, the P3a in passive auditory oddball paradigms is mostly associated with attentional processes, which are not explicitly postulated as part of AP by the two-component model. Further research should be undertaken to determine the influence of attention on pitch processing in AP.

We attempted a direct replication of the original study, still there are some mentionable differences between the original and the replication study that might have influenced the results. While questionnaires on musical experience and the pitch-labeling test were assessed with paper-pencil in the original study, we used online questionnaires and an online pitch-labeling test in the present study. Because our participants underwent an extensive test protocol in the context of the larger AP project spanning several days during which they participated in various (f)MRI and EEG experiments, we tried to keep the travel burden for them as low as possible by providing the opportunity to work on several tests at home. For our statistical analyses, we used the software R instead of SPSS, and we performed Welch’s *t* tests instead of Student’s *t* test because they are more robust for groups with unequal sample sizes (Ruxton, 2006; Delacre et al., 2017). For ANOVAs, we reported generalized eta-squared instead of partial eta-squared as recommended by Bakeman (2005). Like in the original study, groups were defined based on self-report. Contrary to the original study, in our replication study, the non-AP musicians performed above chance in the pitch-labeling test. Accordingly, it could be argued that the groups were less homogenous than in the original study and that this is the reason for the unsuccessful replication. However, because trials in the pitch-labeling test lasted 15 s instead of 5 s, participants probably had enough time to employ RP strategies in our test. It can be expected that highly-trained musicians perform above chance levels when given the opportunity to use RP strategies. For the same reason, it is possible that the pitch-labeling performance of AP musicians was also overestimated. The longer maximal trial duration was due to the online implementation of the pitch-labeling test. In a pilot study, we tested a version with the original trial duration of 5 s, which turned out to be very demanding and difficult to solve even for AP musicians because of the multiple-choice format with 36 answer options. We would recommend future studies to measure reaction times in pitch-labeling tests to be able to better disentangle the effortless and fast AP strategy from the slower RP strategy, or to apply a pitch-labeling test that impedes the usage of RP strategies (e.g., as suggested in Wengenroth et al., 2014). Yet, it still remains unclear which is the best way to objectively identify AP ability and if it is even possible to do so, a question that has been asked frequently and was also discussed in an early influential review on AP (Takeuchi and Hulse, 1993). The authors addressed several methods to quantify AP, ranging from producing tones to different variants of pitch-labeling tests. Up to date, the pitch-labeling tests applied in AP research differ considerably in procedure (e.g., trial duration, answer registration, sine tones/instrumental tones), the number of used tones, and the presentation technique (e.g., online vs lab). Most importantly, no specific cutoff has been established to distinguish AP from non-AP possessors. Thus, in the present study, the pitch-labeling test only served as a validation tool. For group assignment, we relied on self-report since only the participants themselves can judge whether they possess the ability to employ AP strategies. In addition, as demonstrated in the exploratory subgroup analyses, the conclusions of the results remained the same even when just considering participants with the lowest and highest pitch-labeling scores, suggesting that this sample difference between studies did not cause the absence of the AP effect. Similarly, conclusions about the P3a amplitude did not change when just looking at the female participants. Thus, although the original study was less balanced in terms of gender than the present study, the absence of an effect of AP on the P3a amplitude in the present study does not seem to be caused by gender distribution differences between studies. Also, according to current scientific understanding gender differences in neuroscientific cognitive studies are most often due to small sample sizes and should only be interpreted when the influence of hormonal levels was controlled for (Jäncke, 2018). It should also be mentioned that in the present study, the AP and non-AP musicians showed a statistically significant, albeit small in absolute terms (less than three points out of 80 possible points), difference in musical aptitude (AMMA). However, scores are comparable to those reported in the original study, and additional covariance analyses with the AMMA score as covariate showed the same results as the replication analyses.

Finally, it is important to note that a single replication study can never conclusively confirm or disconfirm previous findings. Nevertheless, our results cast reasonable doubt that there is cognitive facilitation in AP during passive tone processing as indicated by the P3a. The more so since our sample was four times the size of the original study, and Bayes factors analyses provided evidence that the proposed effect does not exist. Although it is possible that additional factors we did not control for moderated the effect, we reduced such moderators to a minimum by doing a direct replication. Thus, if an effect of AP on the P3a really exists, its true effect size is probably much smaller than reported in the original study as it is not reliably detectable in a large sample, and its generalizability might be limited.

Considering the large effect size obtained in the original study, the results of the current study demonstrate that only through replications a better estimate of the true effect can be obtained. We believe replications are desirable in science in general and particularly in research fields that are prone to false-positive results and to overestimations of effect sizes due to small samples. Neuroscientific studies often use small samples because of the high financial costs and time-consuming data acquisition and analysis. Collaborative efforts between multiple research groups are suggested as a means to recruit larger sample sizes.

In summary, our direct replication of Rogenmoser et al. (2015) successfully replicated the non-significant results for group differences in the MMN. In contrast, we did not replicate the finding of smaller P3a amplitudes in AP musicians. Taken together, our study does not support electrophysiological differences between AP and non-AP musicians during passive listening. It is conceivable that the different pitch-processing modes of AP and RP can only be reliably distinguished either with more sensitive measures or in more attention-engaging tasks. In more general terms, the results of the present study underline both the importance of replications and of larger sample sizes in neuroscientific research.

## Synthesis

Reviewing Editor: Tatyana Sharpee, The Salk Institute for Biological Studies Decisions are customarily a result of the Reviewing Editor and the peer reviewers coming together and discussing their recommendations until a consensus is reached. When revisions are invited, a fact-based synthesis statement explaining their decision and outlining what is needed to prepare a revision will be listed below. The following reviewer(s) agreed to reveal their identity: Peter Schneider.

This study provides new insights into electrophysiological correlates of superior auditory skills such as absolute and relative pitch perception. Even though the prevalence of absolute pitch is extremely low within the normal population, the existence of such specific neural biomarkers in absolute pitch possessors serves as a model to understand the general mechanisms of pitch perception. The authors are attempting to replicate a previous study that showed smaller evoked P3A potentials in subjects with absolute pitch (AP) compared to subjects with relative pitch (RP). Although they do not find such differences in this study, there is a substantial difference in the music aptitude scores between these two studies. This difference should be discussed and analyzed in ways suggested by Reviewer 2. I would also echo the suggestion from Reviewer 1 on how to discuss the results in relation to prior work.

Reviewer 1:

The manuscript describes a series of experiments to test auditory evoked potentials in an odd-ball paradigm comparing subjects with absolute and relative pitch. The authors are attempting to directly replicate a previous study that showed smaller P3a evoked potential in subjects with absolute pitch. In this replicative study, no such differences were found. Additionally, the authors of the current study use additional statistical testing (Bayesian inference) to show that absent differences is more probably under the null than the alternative hypothesis, i.e. that the absent difference was not due to power issues.

The authors are to be a commended for attempting to replicate a previous study, something that is sadly lacking in published research. The results are an important addition to the literature, as the origin in of AP has long been of interest to auditory scientists. I applaud the use of more sophisticated analytics to try and sort out evidence for absence from absence of evidence. I have a few suggestions that may help clarify the results.

Experimentally, the authors are limited as they are attempting to directly replicate the conditions of a prior study, so I will not make any major comments on experimental design. However, some additional control analyses may be beneficial to address study differences that may give rise to debate after publication.

1. The authors point out that here was increased heterogeneity in the AP vs RP populations with increased performance on pitch identification in the RP. The authors provide a reasonable explanation for this. It might be beneficial to re-run some of the statistics with more homogeneous sub-groups, say perhaps the most extreme 50% in each group.

2. It would be useful to also see the raw average ERPs under standard and deviant conditions, rather than just the difference waveform. This might inform a reader as to possible differences between the current and the previous work (was it a change in the deviant or the standard P3a).

3. You may also consider discussing some other population/subject differences between the two studies, though I doubt they have any effects on outcome differences. For example, the 2015 study had relatively few males (4/16 AP, 2/10 NAP) where the current work is more evenly balanced. I think there is some literature showing larger P300s in females, though not sure how this might have affected the present outcomes (i.e Conroy and Polich, Psychophysiology 2007).

4. This is personal preference, but I find leading the introduction with a discussion about replication a bit aggressive, rather than first starting with a bigger framing of the issues about absolute pitch, as was done in the abstract. I will defer to the authors' judgement about how they feel best to frame their paper, but if it were me I would change the order of the introduction.

Reviewer 2:

It is worthwhile for the community to perform such a replication study and to report and discuss such substantial differences in the outcome. The authors have carefully applied the same design as used in the original study from Rogenmoser et al. 2015.

However I have some comments and questions that should be clarified.

(1) The subjects of the replication study show a significant lower average value of the musical aptitude test (AMMA) in the replication study as compared to the original study (a raw score of 51 out of 80 in the present study as compared to a score of 66 out of 80 in the Rogenmoser study). A total score of 50 in the AMMA test corresponds to a typical result of a lay or amateur musician, whereas a score of 66 indicates a typical result of a professional musician as verified by E. Gordon himself and furthermore in recent research studies (e.g. Schneider et al., Nat Neurosci 2002). Because the electrophysiological response activity of the auditory cortex strongly reflects the musical expertise (see the correlation plots in the above mentioned study between AMMA score and dipole amplitude of ERFs), it could be expected that the MMN and P3a results presented in the replication study may be different if the groups were matched for musical aptitude. I propose to calculate the responses shown in Fig. 1 and Fig. 2 separately for a subgroup with high AMMA score (&gt; 60) compared to a group with lower AMMA score (&lt; 60) and then to check the MMN and P3 results again.

(2) The replication study comprises a large sample that is balanced for gender (50 % each), whereas the Rogenmoser study investigated a sample with a large majority of female subjects. Therefore it would be helpful to check for gender differencs in the present study.

(3) The RP group should better be named ‘non AP group’ (c.f. Wengenroth et al. 2014), because no relative pitch test has been performed in this study. To my experience, most of the musicians have poor RP ability. Therefore it is misleading to speak from RP ability in the absence of AP. (this is also the same problem in many previous AP studies).

(4) The authors should give a reason why the frontal and central electrodes have been pooled to a grand average, and for example not the electrodes above the temporal lobes have been analysed (similar in the Rogenmoser study). As indicated in the discussion, the main generators of MMN are located primarily in the auditory cortex and not in the frontal lobe?

(5) It should be explained why the classification of AP has been done by self-estimation and not by the result of the pitch-labeling test. As seen in the boxplots in Fig. 4 there is a large overlap between the AP and non-AP group that might have a crucial influence on the main EEG results. It would be more objective to classify the AP group according an appropriate cutoff value in the pitch-labeling test as figured out in the Rogenmoser paper.
